# The efficacy and safety of microwave ablation in benign large thyroid nodules (≥4 cm)

**DOI:** 10.1530/ETJ-26-0013

**Published:** 2026-09-15

**Authors:** Hongye Gu, Chaonan Li, Yunhao Luo, Yunjun Liu, Chao Feng, Zhe Song, Jun Luo

**Affiliations:** ^1^ Department of Ultrasound Imaging, Sichuan University affiliated Chengdu Second People's Hospital, Chengdu Second People’s Hospital, West China School of Medicine, Sichuan University, Chengdu, China; ^2^ Ultrasonic Medical Department, Hospital of Chengdu University of Traditional Chinese Medicine, Chengdu, China; ^3^ School of Medical and Life Sciences, Chengdu University of Traditional Chinese Medicine, Chengdu, China; ^4^ Department of Thyroid Surgery, Sichuan Provincial People’s Hospital, University of Electronic Science and Technology of China, Chengdu, China; ^5^ Department of Ultrasound, Sichuan Provincial People’s Hospital, University of Electronic Science and Technology of China, Chengdu, China

**Keywords:** benign large thyroid nodules, microwave ablation, volume reduction rate, MWA

## Abstract

**Objective:**

The objective of this study was to evaluate the safety and efficacy of ultrasound-guided single-session microwave ablation for benign thyroid nodules >4 cm by comparing outcomes with smaller nodules (<4 cm).

**Methods:**

This retrospective single-center study (2016–2023) analyzed 1,107 patients with 1,463 pathologically confirmed benign thyroid nodules stratified by maximum diameter into four groups: ≤20, 20–40, 40–60, and ≥60 mm. Standardized follow-up was performed at 1, 3, 6, 12, and 24 months post-microwave ablation and then annually. Volume reduction ratio, maximum diameter reduction ratio, recurrence rate, and complications were assessed.

**Results:**

The cohort (127 males and 980 females) had a mean age of 45.8 ± 12.7 years and a mean maximum nodule diameter of 28.9 ± 14.6 mm. Complication rates significantly increased with nodule size (2.59, 5.55, 13.36, and 16.67%, respectively; *P* < 0.001). Technical efficacy exceeded 90% across all groups (*P* > 0.05). Volume reduction ratio improved significantly over time across all groups, with statistical differences at 1, 6, 12, and ≥24 months (all *P* < 0.001) but not at 3 months (*P* = 0.085). The ≤20 mm group achieved the highest volume reduction ratio at ≥24 months (96% ± 14%).

**Conclusion:**

Ultrasound-guided single-session microwave ablation is safe and effective for large benign thyroid nodules, providing significant volume reduction with acceptable complication profiles while reducing treatment burden.

## Introduction

Thyroid nodules are a common clinical finding, with high-resolution ultrasound detecting them in 20–76% of individuals, over 90% of which are benign (1, 2). Most pathologically confirmed benign nodules only require active surveillance, and asymptomatic, non-growing nodules generally do not require intervention (3, 4, 5). However, nodules exceeding 4 cm in diameter exhibited an increased risk of capsular or vascular invasion (which transforms a follicular adenoma into follicular carcinoma) (6). Therefore, nodules exceeding 4 cm in diameter or those causing compressive symptoms or cosmetic concerns usually warrant treatment (7, 8).

Although surgical resection remains the conventional approach (3, 4, 5), it is limited by cost, general anesthesia, potential thyroid dysfunction requiring lifelong hormone replacement, complications, and cosmetic impact (9, 10, 11). In recent years, non-surgical minimally invasive therapies have gained increasing attention (12, 13). Thermal ablation has emerged as a first-line alternative to surgery for benign thyroid nodules. However, most evidence focuses on radiofrequency ablation, with limited data on the efficacy and safety of microwave ablation (MWA), particularly for large nodules (≥4 cm or volume >20 mL), where its role remains controversial.

Compared with radiofrequency ablation, MWA generates higher and more sustained intratumoral temperatures, creates larger ablation zones, shortens procedure time, and is less affected by tissue conductivity (14, 15, 16, 17). While these characteristics suggest advantages for large nodules, concerns remain regarding complications such as periprocedural edema, thermal injury to adjacent tissues, and airway compromise due to greater heat diffusion. Although MWA has proven safe and effective for small benign nodules (18, 19, 20), robust clinical evidence for its use in large benign nodules (≥4 cm) is still lacking (19). This study aims to further evaluate the efficacy and safety of microwave ablation for large benign thyroid nodules.

## Materials and methods

A retrospective analysis was performed on patients with pathologically or cytologically confirmed benign thyroid nodules meeting ablation criteria at Sichuan Provincial People’s Hospital (2016–2023). All patients received comprehensive information on treatment options and provided written informed consent prior to treatment. The inclusion criteria were as follows: i) ultrasound suggesting benign nodules, with benign nature verified via fine-needle aspiration (FNA) cytology (Bethesda category II) or preoperative core needle biopsy (CNB) histopathological examination; ii) no history of childhood neck radiotherapy; iii) fully informed patient preference for minimally invasive interventional therapy over surgery or clinical observation; iv) patients with progressively enlarging nodules causing compressive symptoms, cosmetic concerns, significant psychological distress impacting daily life, and refusing surgery; and v) a minimum follow-up duration of six months. The exclusion criteria were as follows: i) unclear pathology or confirmed malignancy: FNA biopsy revealing Bethesda category I, III, IV, V, or VI cytology or CNB demonstrating indeterminate or malignant histopathologic results; ii) severe coagulation dysfunction; iii) significant impairment of vital organ function; iv) predominantly cystic nodules (>90% cystic component); and (v) incomplete clinical data. The specific process is shown in Fig. 1. Patients were stratified into four groups based on the ultrasound-measured maximum nodule diameter: ≤20, 20–40, 40–60, and ≥60 mm. The occurrence and recovery of complications during and after MWA, as well as the volume reduction rate (VRR), maximum diameter reduction rate (MDRR), and recurrence rate at 1, 3, 6, 12, and 24 months and longer after the operation, were collected. These data were analyzed to compare the impact of nodule size on therapeutic outcomes and evaluate the effectiveness and safety of ultrasound-guided microwave ablation for large benign thyroid nodules. This study was approved by the Medical Ethics Committee of Sichuan Academy of Medical Sciences Sichuan Provincial People’s Hospital (Approval No. 20210302), and all enrolled patients provided written informed consent.

**Figure 1:**
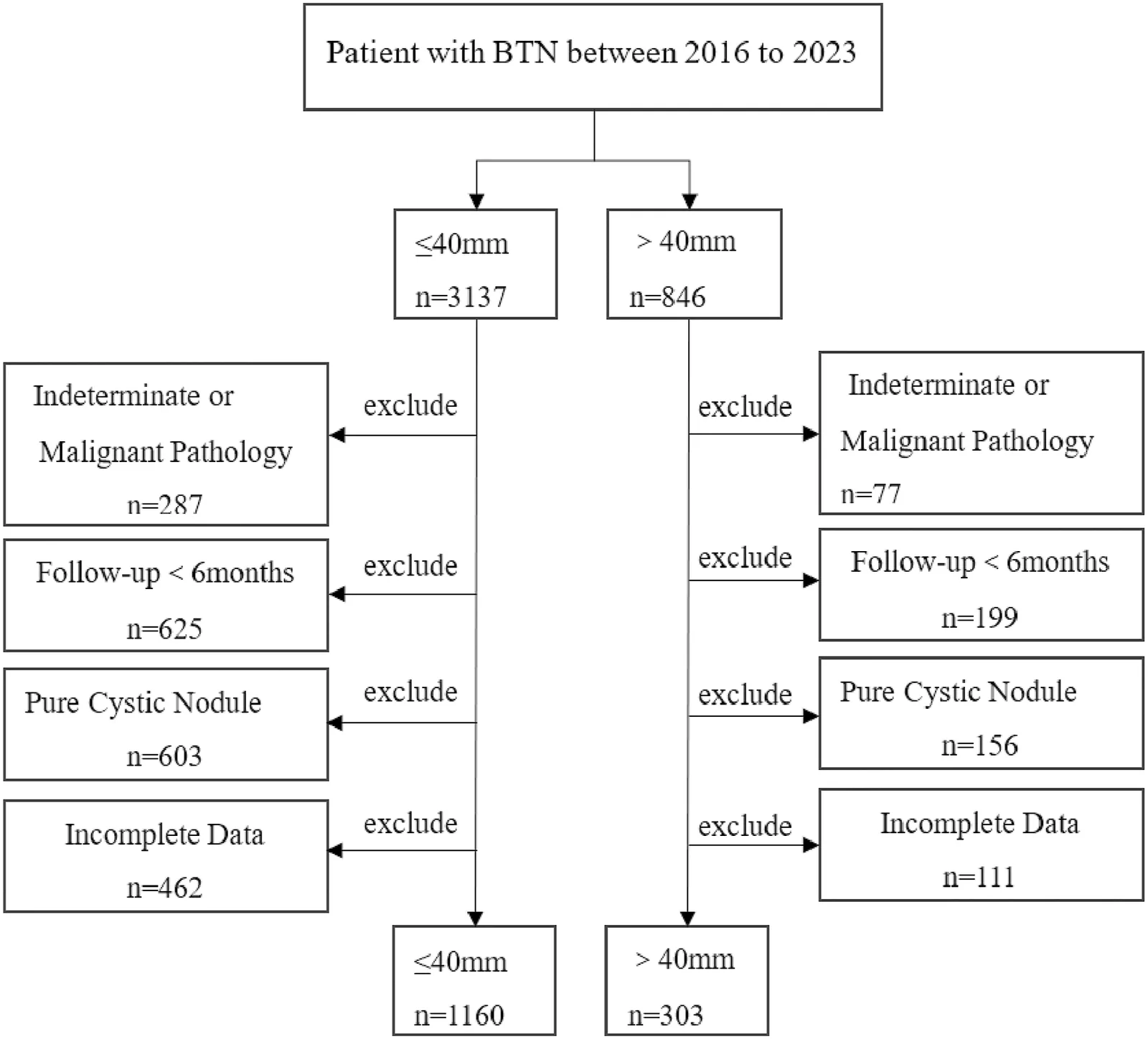
Study flow chart. BTN, benign thyroid nodule.

### Preoperative assessment

All patients underwent routine ultrasound examination of the thyroid and cervical lymph nodes, ultrasound-guided FNA or core needle biopsy (CNB), and laboratory tests before the operation. The location, size, composition, and blood flow signals of thyroid nodules were evaluated and recorded. The laryngeal status was subjectively assessed by the ablation doctor based on the specific condition of the patient. Laryngoscopy should be performed for patients with a high-risk medical history or a history of neck surgery. Laboratory tests include thyroid function (at least including TSH, FT3, and FT4), complete blood count, and coagulation function. All ultrasound examinations, CNB, and FNA biopsy procedures were performed by doctors with more than five years of experience in ultrasound and interventional procedures.

### Intraoperative procedures

All MWA procedures were performed by a physician with 10 years of experience in thyroid ultrasound, 5 years in thyroid FNA, and over 50 thyroid nodule ablation cases.

Preoperatively, contrast-enhanced ultrasound (SonoVue) was used to assess nodule enhancement patterns, guiding ablation planning and serving as a postoperative baseline.

The patient was positioned supine with the neck extended and received local anesthesia with 1% lidocaine. Hydrodissection was conducted by injecting sterile normal saline between the thyroid lobe with the target nodule and adjacent vital structures, creating a protective isolation zone of approximately 5 mm, which was maintained by continuous slow saline infusion as required. Based on the nodule’s characteristics, size, and anatomical location, the optimal ablation power (15–50 W, mean: 29.7 W) and ablation duration (5–2,230 s, mean: 241.1 s) were determined. Following local anesthesia, a disposable sterile 14 G × 10 cm microwave ablation needle with a 1-mm active tip was percutaneously punctured into the central region of the nodule. Ablation was performed using the moving-shot technique, ensuring that the hyperechoic gas produced during the procedure fully covered the entire nodule and extended at least 1–2 mm beyond its original boundaries, with the mean ablative energy applied at approximately 3.4 kJ per milliliter of nodule volume. Ablation was terminated once the aforementioned coverage was attained. Contrast-enhanced ultrasound (CEUS) was performed at 5 min post-ablation to evaluate the completeness of ablation. In cases where residual enhancement within the nodule indicated incomplete ablation, additional ablative treatment was administered under real-time CEUS guidance. The surgical procedure was formally concluded only after CEUS confirmed the absence of any intranodular enhancement.

### Postoperative follow-up

Ultrasound follow-up was performed at 1, 3, 6, 12, and 24 months after MWA, with additional annual examinations beyond that point. During each follow-up visit, thyroid ultrasound was conducted to document the size of the ablated zone and assess the presence of blood flow within the ablation area using color Doppler flow imaging (CDFI). CEUS was employed when necessary to evaluate postoperative recovery and detect potential recurrence. In cases where recurrence was identified, secondary ablation or surgical intervention was considered based on clinical evaluation. All major and minor complications and side effects were systematically recorded. All ultrasound examinations were performed by physicians with more than 10 years of experience in thyroid and cervical lymph node sonography.

### Main evaluation indicators and evaluation criteria

The primary evaluation indicators in this study included primary technical success rate, technical efficacy rate, VRR, MDRR, recurrence rate, complication rate, and recovery status. The specific criteria for each indicator were defined as follows: i) Primary technical success: complete ablation of the target nodule in a single session, with no enhancement observed on immediate post-procedural CEUS, and no premature termination of the procedure due to patient-related or other factors. ii) Technical efficacy (21): a VRR ≥ 50% at the 12-month follow-up. iii) VRR: calculated as VRR = ((Vinitial – Vfinal)/Vinitial) × 100%. The volume of the nodules was calculated based on the diameter using the following formula: V = πabc/6 (where ‘V’ is the volume, ‘a’ is the maximum diameter, and ‘b’ and ‘c’ are the other two perpendicular diameters). iv) MDRR: calculated as MDRR = ((ainitial – afinal)/ainitial) × 100%. v) Recurrence: defined as the emergence of suspicious regenerative nodules within the ablation zone on two-dimensional ultrasound, accompanied by internal vascular signals on CDFI or contrast agent filling on CEUS, or a reduction in VRR compared with the previous follow-up examination. vi) Complications: classified according to the Society of Interventional Radiology (SIR) guidelines (22, 23). Major complications: events that may threaten life, cause severe disability, prolong hospitalization, or require intensive intervention (e.g. persistent voice changes, nodule rupture, and injury to adjacent vital structures). Voice changes were evaluated based on patient reports, laryngeal ultrasound, and laryngoscopy. Minor complications: all other complications not classified as major complications (e.g. hematoma and skin burn). vii) Side effects: self-limiting symptoms not requiring therapeutic intervention (e.g. mild pain and transient choking sensation). viii) Asphyxia: a critical condition involving airway obstruction or respiratory dysfunction due to periprocedural complications, leading to hypoxemia and hypercapnia. Potential causes included direct tracheal compression, recurrent laryngeal nerve injury, laryngospasm, laryngeal edema, or improper patient positioning. ix) Hematoma: defined as any sonographically visible collection of blood within or around the thyroid gland that resulted in incomplete ablation (24). x) Pain (complication): pain persisting for more than three days post-ablation despite analgesic treatment and leading to incomplete ablation. Other pain presentations were considered side effects. Tolerable post-procedural discomfort was not classified as a complication or side effect (24). xi) Dyspnea (side effect): subjective breathing discomfort accompanied by objective abnormalities in respiratory rate, depth, or rhythm due to procedure-related factors, not yet meeting criteria for asphyxia but requiring monitoring for potential progression. xii) Statistical unit: complication rates were calculated per patient. Primary technical success, recurrence, technical efficacy, and follow-up outcomes were calculated per nodule. xiii) Ablation energy per unit volume: energy delivered per unit volume of nodule = Time (s) × Power (W)/Volume (cm^3^).

### Statistical analysis

Data were analyzed using SPSS software (version 25.0). Categorical variables are presented as numbers and percentages (*n*, %). The Kruskal–Wallis test was applied for comparing non-normally distributed continuous variables and ordinal data across groups. Comparisons of categorical variables between groups were performed using the Pearson chi-square test or the linear-by-linear association chi-square test; Fisher’s exact test was used when appropriate. A two-sided *P*-value of less than or equal to 0.05 was considered statistically significant.

## Results

### Patient characteristics

A total of 1,107 patients with 1,463 nodules were included, comprising 127 males and 980 females. The mean age was 45.8 ± 12.7 years (range: 11–91 years). The mean maximum nodule diameter was 28.9 ± 14.6 mm (range: 2.8–90.0 mm), and the mean follow-up duration was 19.1 ± 13.6 months (range: 6–90 months). Based on the maximum nodule diameter, patients were categorized into four groups: ≤20, 20–40, 40–60, and ≥60 mm. Baseline characteristics of the patients are presented in Table 1.

**Table 1:** Baseline characteristics of patients and nodules.

Characteristics	≤20 mm group	20–40 mm group	40–60 mm group	>60 mm group
Age (years)	45.4 ± 12.2	45.7 ± 12.5	45.7 ± 13.1	48.8 ± 12.7
Sex				
Male	27	56	114	10
Female	166	575	34	26
Total	193 (17.4%)	631 (57.0%)	247 (10.3%)	36 (3.3%)
Nodule location				
Left lobe	200	336	130	20
Right lobe	208	373	133	17
Isthmus	27	16	2	1
Maximum diameter (mm)	12.1 ± 4.8	30.1 ± 5.4	47.0 ± 5.1	69.0 ± 7.3
Volume (mL)	1.2 ± 1.3	13.3 ± 8.3	43.7 ± 22.1	128.4 ± 59.6
Average ablation power (W)	28.6 ± 3.0	29.5 ± 3.2	31.1 ± 3.5	33.0 ± 3.3
Average ablation time (s)	64.8 ± 64.3	221.9 ± 167.3	491.4 ± 294.5	815.1 ± 431.5
Average ablation energy per unit volume of nodule (kJ/mL)	9.3 ± 21.5	1.1 ± 0.9	0.7 ± 0.4	0.5 ± 0.3
Average follow-up duration (months)	19.7 ± 13.3 (1–79)	18.7 ± 13.2 (1–90)	19.1 ± 14.3 (1–80)	20.3 ± 15.1 (1–60)
No. of patients per time point				
6 months	86	305	117	13
12 months	111	372	144	21
24 months	54	138	55	11
36 months	27	92	29	3
48 months	9	32	17	3
60 months	6	9	7	1
≥72 months	2	3	2	0

### Safety analysis

Among 1,107 patients who underwent ablation, the incidence of major complications was as follows: choking in 0.09% (*n* = 1), vocal changes in 3.25% (*n* = 36), nodule rupture with abscess formation in 0.27% (*n* = 3), and parathyroid injury in 0.09% (*n* = 1). Minor complications included bleeding or hematoma in 1.17% (*n* = 13), vomiting in 1.08% (*n* = 12), and skin burn in 0.09% (*n* = 1). Side effects consisted of pain in 1.17% (*n* = 13), vasovagal reaction in 0.45% (*n* = 5), choking cough in 1.09% (*n* = 12), and dyspnea in 0.54% (*n* = 6). All complications resolved within 1–9 months postoperatively. A significant difference was observed in complication rates among nodule size groups (*P* < 0.001), with larger nodules associated with higher complication rates.

The primary technical success rate exceeded 95% across all groups, with smaller nodules exhibiting significantly higher rates of complete initial ablation (*P* < 0.001). The technical efficacy rate was above 90% in all groups, with no statistically significant differences observed among them. Postoperative recurrence rates increased significantly with a larger nodule diameter (*P* < 0.05). Safety outcomes are summarized in Table 2.

**Table 2:** Safety of MWA treatment.

	≤20 mm group		20–40 mm group		40–60 mm group		>60 mm group		*X^2^*	*P*
	Rate, %	*n*	Rate, %	*n*	Rate, %	*n*	Rate, %	*n*		
Complication rate	2.59	193	5.55	631	13.36	247	16.67	36	27.770	<0.001
Primary technical success rate	100	435	99.54	725	99.62	265	97.37	38	9.029	0.029
Effective ablation rate	94.08	227	91.61	310	97.67	129	95.0	20	5.765	0.124
Recurrence rate	1.73	435	3.17	725	8.10	265	8.33	38	16.018	0.001

### Validity analysis

During the follow-up period, the changes in VRR and MDRR at 1, 3, 6, 12, and 24 months or more after surgery in each group are presented in Fig. 2.

**Figure 2:**
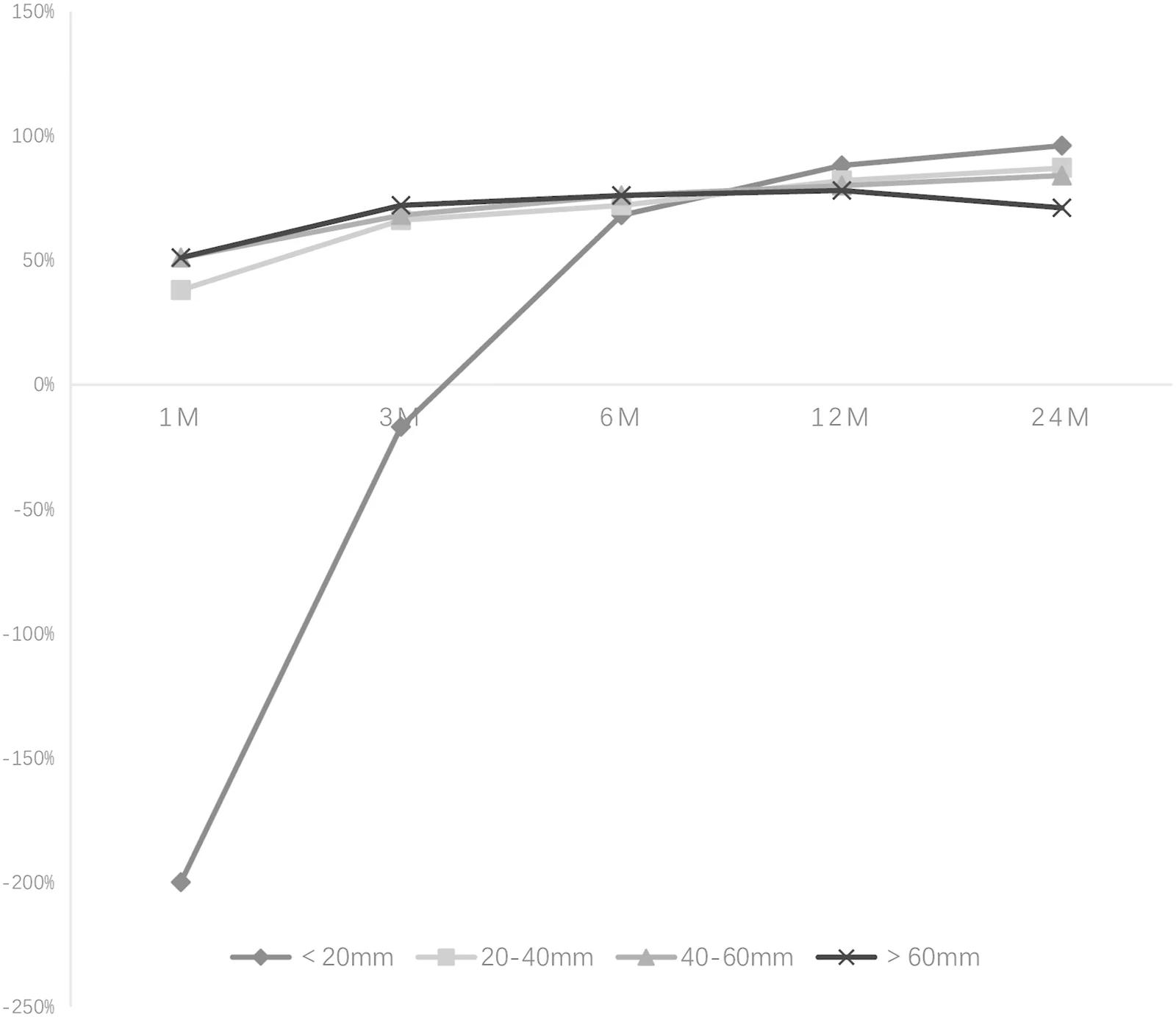
VRR and MDRR at each time point.

Both the maximum diameter and volume of the nodules showed a significant reduction at 3 months post-ablation and beyond in all groups. The VRR progressively increased with extended follow-up, indicating effective and sustained volume control across all four groups following the procedure.

The number of patients in each group at each follow-up time point after surgery and MDRR are shown in Table 3. Comparison of MDRR among the groups at 1, 3, 6, 12, and ≥24 months after MWA showed no statistically significant differences at the 3- and 6-month follow-ups. However, significant differences in MDRR were observed among the groups at 1, 12, and ≥24 months. The 40–60 mm group showed a better volume reduction at the 1-month follow-up, while the ≤20 mm group exhibited superior MDRR outcomes at 12 months and beyond.

**Table 3:** Comparison of maximum diameter reduction rates (MDRRs) for different nodule sizes at different time points.

Time point	≤20 mm group		20–40 mm group		40–60 mm group		>60 mm group		*X^2^*	*P*
	MDRR, %	*n*	MDRR, %	*n*	MDRR, %	*n*	MDRR, %	*n*		
1 month	−14 ± 74	107	21 ± 20	218	25 ± 11	175	25 ± 11	31	65.074	<0.001
3 months	29 ± 50	114	37 ± 16	232	38 ± 15	93	34 ± 12	13	4.827	0.185
6 months	51 ± 40	185	44 ± 22	341	43 ± 16	129	41 ± 12	13	6.802	0.078
12 months	67 ± 36	227	52 ± 24	425	48 ± 19	152	45 ± 15	22	57.097	<0.001
24 months	85 ± 27	230	63 ± 27	320	55 ± 19	115	52 ± 22	20	134.141	<0.001

The number of patients in each group at each follow-up time point after surgery and VRR are shown in Table 4. The VRRs were compared among the groups. No statistically significant difference in VRR was observed at the 3-month follow-up. However, significant differences were detected at 1 month, 6 months, 12 months, and ≥24 months after MWA. The 40–60 mm group exhibited a better volume reduction at 1 month, whereas the ≤20 mm group showed superior VRR outcomes at all subsequent time points.

**Table 4:** Comparison of volume reduction rates (VRRs) for different nodule sizes at different time points.

Time point	≤20 mm group		20–40 mm group		40–60 mm group		>60 mm group		*X^2^*	*P*
	VRR, %	*n*	VRR, %	*n*	VRR, %	*n*	VRR, %	*n*		
1 month	−199 ± 671	104	36 ± 41	212	50 ± 26	95	37 ± 67	17	46.729	<0.001
3 months	8 ± 209	102	67 ± 23	219	69 ± 17	89	73 ± 17	13	2.488	0.477
6 months	75 ± 41	162	73 ± 33	301	75 ± 12	121	76 ± 12	14	21.928	<0.001
12 months	89 ± 23	169	82 ± 19	310	81 ± 14	129	79 ± 13	20	87.228	<0.001
24 months	96 ± 14	206	86 ± 19	261	85 ± 17	107	78 ± 13	20	176.30	<0.001

Figure 3 presents a clinical case, including the ultrasound images of the patient’s nodule before microwave ablation, during the procedure, and immediately after surgery, as well as the two-dimensional ultrasound images from postoperative follow-up. The ablated nodule in this patient showed a gradual trend of absorption and reduction in size with the passage of follow-up time.

**Figure 3:**
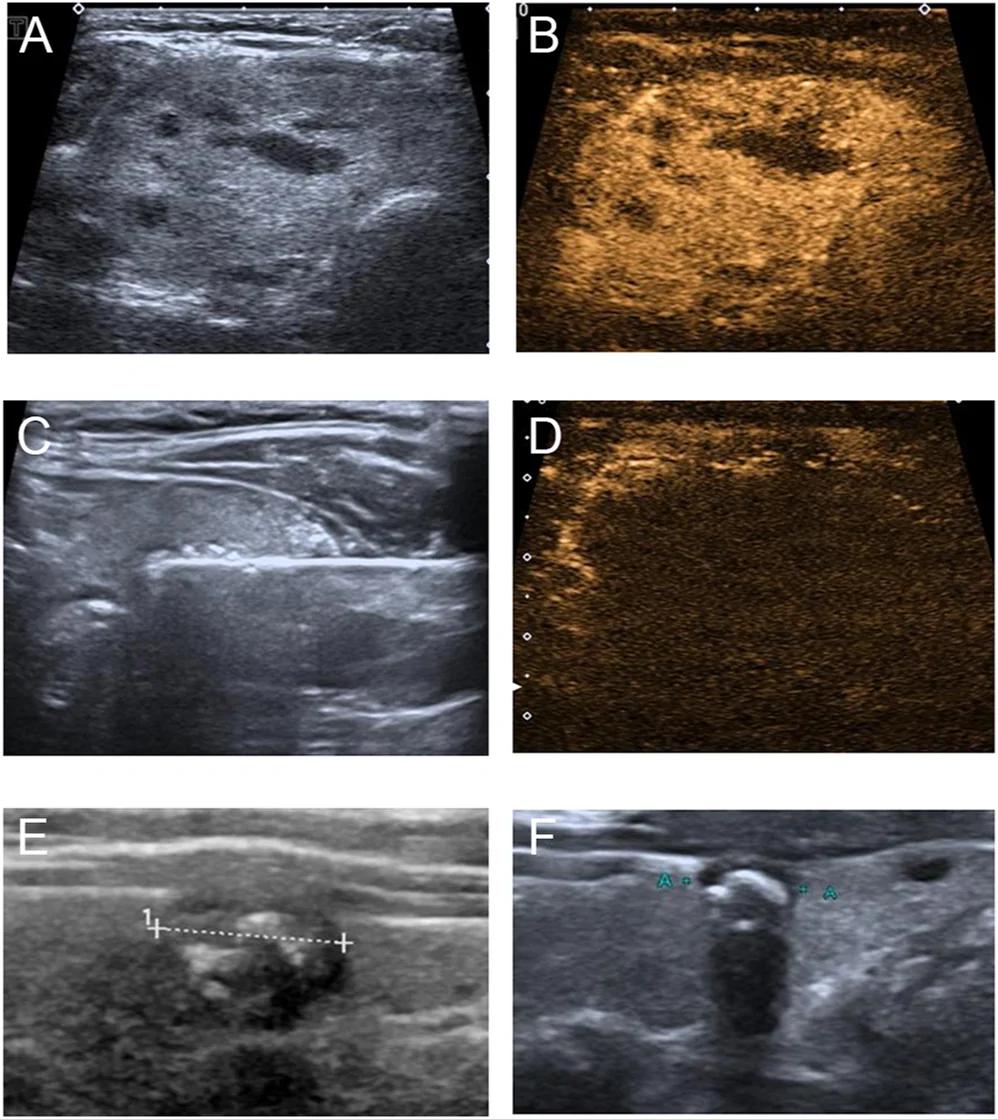
Case report. Ultrasound images in a 51-year-old female patient with a large benign thyroid nodule (size: 52.3 × 40.9 × 28.7 mm). (A) Routine ultrasound shows a single nodule in the right lobe. (B) Pre-ablation contrast-enhanced ultrasound demonstrates hyperenhancement during the arterial phase. (C) Percutaneous microwave ablation was performed using the moving-shot technique. (D) Post-ablation contrast-enhanced ultrasound reveals no enhancement in the original lesion. (E) Eight months after MWA (size: 15 × 9 × 10 mm). (F) Two years after MWA (size: 8 × 7 × 7 mm).

## Discussion

Currently, there is no universally accepted definition of ‘large’ benign thyroid nodules. In previous studies, this term has been variably defined according to maximum diameter (e.g. ≥25–50 mm) or nodule volume (e.g. ≥20–30 mL (25, 26, 27, 28, 29)), mainly for research stratification and treatment planning rather than for pathological classification. However, larger nodules may be more likely to cause compressive symptoms or cosmetic concerns, and their size may also influence the technical complexity and efficacy of ultrasound-guided ablation. For benign thyroid nodules requiring intervention, treatment decisions should be based on a combination of clinical symptoms, cosmetic problems, cytological and ultrasonographic findings, patient preference, and procedural feasibility. Although surgery has traditionally been the standard treatment for symptomatic benign nodules, it is associated with several drawbacks, including hospitalization costs, anesthesia-related burden, risk of postoperative hypothyroidism, surgical complications, and cosmetic issues (9, 10, 11).

Thermal ablation has emerged as a widely adopted minimally invasive treatment modality for benign thyroid nodules. Numerous studies have demonstrated its efficacy in significantly reducing nodule volume (18, 30, 40). Compared with surgery, thermal ablation offers superior cosmetic outcomes and fewer complications. The Italian guidelines for thyroid surgery underscore the clinical significance of thermal ablation (31), and several major guidelines endorse it as a therapeutic option for thyroid nodules (1, 32). While most previous research has focused on radiofrequency ablation (RFA), fewer studies have evaluated the efficacy and safety of MWA. Compared with RFA, MWA achieves higher sustained intratumoral temperatures, generates larger ablation zones, requires shorter procedure times, and exhibits less dependence on tissue electrical conductivity (14, 15, 16, 17). These properties suggest potential therapeutic advantages for large nodules, although MWA also carries a theoretical risk of greater thermal injury to surrounding tissues and procedure-related complications. Concerns regarding postoperative edema, tracheal compression, and potential asphyxia necessitate careful patient selection. Therefore, this study adopted a maximum nodule diameter of ≥40 mm as the classification criterion to evaluate the safety and efficacy of MWA for large benign thyroid nodules.

In the study by Du *et al.*, a VRR of 96.9 ± 2.5% was achieved at 48 months after microwave ablation in 148 benign thyroid nodules ≥2 cm (33). An Italian study reported a VRR of 81.6 ± 15.8% at 6 months post-microwave ablation in 150 benign thyroid nodules ≥2.5 cm (34). A multicenter study by Cang *et al.* demonstrated a VRR of 76.09 ± 2.27% at 12 months after microwave ablation in 87 benign thyroid nodules ≥4 cm (41). Luo *et al.* observed a VRR of 86.6 ± 14.7% at 12 months following ablation in 37 benign thyroid nodules with a volume ≥30 mL (21). Compared with previous studies, our study included a larger patient cohort, had a longer follow-up duration, and conducted comparative analyses among nodule subgroups, while achieving consistent outcomes. In our study, the mean VRR in the large-nodule group reached 81 ± 14% after one year of follow-up, with a technical efficacy rate of 97.7%. All complications were transient and resolved completely. These results collectively demonstrate that microwave ablation is a safe and effective treatment for large benign thyroid nodules, achieving a significant volume reduction with few and mild complications. For particularly large nodules, fractionated ablation or palliative ablation may be considered to optimize therapeutic outcomes while minimizing the risk of complications.

Consistent with previous studies (33, 35), the ≤20 mm group generally exhibited better volume reduction outcomes at 12 months and beyond. However, this group showed no significant advantage in the volume reduction rate (VRR) at the 1- and 3-month follow-ups compared with other groups. This phenomenon may be attributed to the relatively large thermal ablation zone produced by MWA. During the procedure, smaller nodules are more susceptible to inadvertent ablation of adjacent normal thyroid tissue. Consequently, the non-perfused area observed on post-operative imaging often exceeds the original nodule volume, leading to a transient increase in the apparent ablation zone size. As a result, at the 1-month follow-up, some patients may exhibit an ablated area that appears larger than the pre-operative nodule volume.

Patients with nodules ≤20 mm are typically asymptomatic and without cosmetic concerns. Ablation treatment in this group is often initiated due to patient request – either during the concurrent treatment of larger nodules (>20 mm) or due to anxiety-driven preference for intervention. Targeting such small nodules for ablation may constitute overtreatment. Furthermore, the prognostic outcomes indicate that smaller nodules achieved better long-term nodule volume shrinkage outcomes and may even undergo complete resolution. In this study, with follow-up extending to 24 months or longer, 81% of the nodules in the ≤20 mm group eventually achieved complete absorption and disappearance.

In this study, the recurrence rate in the large-nodule group ranged from 8.1 to 8.33%. Postoperative recurrence increased with a larger nodule diameter, consistent with previous studies (36, 37, 38). This trend may be attributed to several technical and biological factors. Due to their higher volume, large nodules often require multipoint and repeated ablations to achieve full coverage. Needle trajectory limitations and larger deflection angles can further contribute to incomplete ablation in certain areas.

Additionally, uneven energy distribution during ablation may result in significant temperature disparities between the center and the periphery of the nodule. While the central zone often achieves complete necrosis due to high temperatures, the peripheral zone – where heat is dissipated by surrounding blood flow and fluid – may retain viable tissue. Moreover, attenuation of microwave or radiofrequency energy in deeper tissue regions can prevent effective thermal inactivation, resulting in incomplete ablation.

Large nodules also frequently possess a richer blood supply (42), which can cool the marginal tissue during ablation and allow residual cells to survive. These remaining active cells may subsequently lead to recurrence. Furthermore, although CEUS was used intraprocedurally as a criterion for complete ablation, it serves only as a relative gold standard in clinical practice and does not guarantee long-term prognostic success. Nevertheless, failure to use immediate post-ablation CEUS evaluation is associated with higher risks of residual and recurrent lesions (42).

In previous studies, the ablation of large thyroid nodules mostly adopted a multi-session approach, typically involving 2–3 treatments (21, 39). In contrast, this study primarily employed a single-session ablation protocol, with a palliative ablation rate of only 0.71%. Although the postoperative recurrence rate observed here was higher than that reported in previous studies using multi-session ablation, the types and incidence of complications were comparable, showing no statistically significant difference. Moreover, the single-session protocol helped reduce treatment costs for patients and improved treatment compliance.

The types and incidence of complications observed in this study are consistent with those reported in other similar studies (34, 39, 40). Complication rates varied significantly across groups depending on nodule size, with larger nodules associated with both a higher frequency and greater severity of complications. The ablation of larger nodules also places greater technical demands on the operator. Compared with smaller nodules, larger ones require higher energy outputs and longer ablation times to achieve complete necrosis. Furthermore, given their proximity to critical surrounding tissues and organs, larger nodules exhibit a more pronounced thermal focusing effect during treatment. This necessitates more extensive fluid isolation for effective protection of adjacent structures. Inadequate technique or uneven fluid distribution may compromise this protective effect, thereby increasing the risk of complications.

Thermal ablation remains an effective treatment option for larger nodules, demonstrating favorable prognostic outcomes. Although the incidence of complications increases with nodule size, the complication rate for nodules < 60 mm remains relatively low, ranging from 6.1 to 13.2% (40, 41). Many of these complications can be mitigated through standardized operative procedures, operator experience, and meticulous technique. Previous studies have indicated that targeted ablation of feeding arteries can reduce the risk of bleeding and hematoma formation, yielding improved clinical outcomes (42). It should be noted that this study included a limited number of nodules > 60 mm. As a result, the estimated complication rate in this subgroup is associated with relatively wide confidence intervals. Further studies with larger sample sizes are needed to more accurately characterize complication risks in this population.

Both previous studies and the current research consistently demonstrate that smaller nodules are associated with more favorable ablation outcomes, including fewer and less severe complications, lower technical demands on the operator, and a higher rate of complete postoperative resolution. However, incomplete absorption of the ablated lesion – particularly when accompanied by suspicious sonographic features during follow-up – can pose diagnostic challenges for clinicians and cause unnecessary anxiety for patients. Therefore, it is essential to critically evaluate the indications for ablating benign thyroid nodules. Intervention might reasonably be considered when nodules reach a certain size, cause compressive or cosmetic symptoms, or exhibit significant growth during monitoring – rather than based on size alone.

Although surgery remains a widely used and well-established treatment, accumulating long-term follow-up studies indicate that thermal ablation is an effective modality for benign thyroid nodules and is increasingly recognized as a first-line alternative to surgery. A meta-analysis by Xu *et al.* demonstrated that thermal ablation leads to a significant and sustained volume reduction in benign thyroid nodules (43). Previous comparative studies have evaluated the efficacy and safety of thermal ablation versus conventional surgery for nodules larger than 4 cm. After 12 months of follow-up, microwave ablation was shown to be a safe and effective treatment for such nodules. Compared with traditional thyroid lobectomy, it offers faster recovery, fewer complications, better preservation of thyroid function, and superior cosmetic outcomes (26). This study further supports the safety and efficacy of microwave ablation for large benign thyroid nodules, adding to the growing body of clinical evidence.

The primary therapeutic objectives of thermal ablation for benign thyroid nodules include nodule volume reduction, alleviation of compressive symptoms, and cosmetic improvement, and complete ablation is not a mandatory clinical requirement. In the present study, complete microwave ablation was performed on all enrolled large benign thyroid nodules to maximize the volume reduction rate and achieve sustained long-term nodule shrinkage. However, blind pursuit of full circumferential ablation significantly raises the risk of injury to vital adjacent anatomical structures, such as the recurrent laryngeal nerve and parathyroid glands, thereby increasing the overall incidence of ablation-related complications. When developing individualized ablation protocols for large thyroid nodules in clinical practice, clinicians must fully weigh the balance between optimal volumetric efficacy and procedural safety. For nodules with a diameter ≥4 cm, operators should comprehensively evaluate the anticipated shrinkage effect alongside procedure-associated risks when determining ablation strategies.

### Limitations

This study has several limitations. First, as a single-center retrospective analysis, it is prone to selection bias and lacks broad representativeness, warranting validation of the findings via multicenter prospective studies. Second, despite the relatively large patient cohort, the rate of regular complete follow-up was suboptimal, which may lead to attrition bias and compromise the accuracy and generalizability of statistical results. Third, the long-term prognosis of ablated large benign thyroid nodules requires prolonged observation, and additional long-term follow-up data are needed to confirm the findings. Fourth, prior evidence has indicated that nodule composition may affect ablation efficacy, yet this factor was not analyzed or controlled in the study, potentially introducing confounding effects. Another prominent limitation of this study is the high loss-to-follow-up rate among patients beyond the 6-month follow-up window, which prevents us from calculating the long-term nodule regrowth rate. Most patients opted to undergo ultrasound examinations at local hospitals closer to their residences due to long travel distances, relieved compressive symptoms, and satisfactory cosmetic outcomes, leading to incomplete long-term data collection. Further prospective studies with extended and standardized follow-up protocols are required to characterize the long-term patterns of nodule regrowth and recurrence following microwave ablation for large benign thyroid nodules.

## Declaration of interest

There is no conflict of interest that could be perceived as prejudicing the impartiality of the research reported.

## Funding

This research did not receive any specific grant from any funding agency in the public, commercial, or not-for-profit sector.

## Author contribution statement

HG and JL conceived and designed the study and wrote the manuscript. CL developed the methodology and conducted the experiments. YL and YL performed data analysis and visualization. CF and ZS supervised the research and administered the project.
